# Quantum-inspired K-nearest neighbors classifier for enhanced printer source identification in forensic document analysis

**DOI:** 10.1038/s41598-025-86558-y

**Published:** 2025-02-03

**Authors:** Saad M. Darwish, Raad A. Ali, Adel A. Elzoghabi

**Affiliations:** Department of Information Technology, Institute of Graduate Studies and Research, Alexandria University, 163 Horreya Avenue, El Shatby, P.O. Box 832, Alexandria, 21526 Egypt

**Keywords:** Printer forensics, Document source identification, Quantum-inspired computing, Feature modeling, Classification, Computer science, Information technology, Software

## Abstract

Document source identification in printer forensics focuses on determining the source printer of a document by analyzing characteristics such as printer model, serial number, defects, or unique artifacts. This is crucial in forensic investigations involving counterfeit documents or anonymous threats. However, identifying consistent patterns across different printers remains challenging, especially when perpetrators attempt to obscure these artifacts. Machine learning models in this field must identify discriminative features that differentiate printers while minimizing noise. In particular, choosing an appropriate distance metric for K-Nearest Neighbors (KNN) classifiers is critical and requires experimentation. This study proposes a quantum-inspired approach to improve KNN’s performance in printer source identification. By exploring alternative number of neighbors (*K*), quantum-inspired computing can optimize feature space calculations, even in noisy conditions. This allows the system to iteratively refine and select the optimal K value based on classification performance, ensuring that the best K is identified for the specific dataset and task. The system utilizes the Grey Level Co-occurrence Matrix (GLCM) for feature extraction, which is robust to changes in rotation and scale. Experimental results demonstrate that the Quantum-inspired KNN (QKNN) classifier outperforms classical KNN, achieving higher accuracy in identifying subtle printing artifacts, even under variable conditions.

## Introduction

Printer forensics involves the analysis of printed documents to identify and attribute characteristics to a specific printer or class of printers. This branch of forensic science focuses on examining physical and digital evidence to trace the origin of a document, detect forgeries, and establish authenticity. Document source identification in printer forensics involves techniques and methods used to trace a printed document back to the specific printer that produced it. This process is essential in various applications such as criminal investigations, fraud detection, and authentication of important documents^1^. Document source identification in printer forensics encounters several significant challenges that can impact the accuracy and reliability of forensic conclusions. These challenges stem from both technical and procedural issues, and overcoming them is crucial for effective forensic analysis^2,3^. These challenges including (1) Intra-Printer Variability: Even the same printer can produce variations in output due to changes in ink or toner levels, paper type, or environmental conditions. This variability can complicate the identification process. (2) Inter-Printer Variability: Different models or even different units of the same model can exhibit unique characteristics. Identifying a document’s source becomes more complex when accounting for the wide range of printers available. (3) Printer Aging: Printers undergo wear and tear over time, which can change their printing characteristics. This evolution can make it difficult to match documents printed at different times^1,2^.

**Fig. 1:**
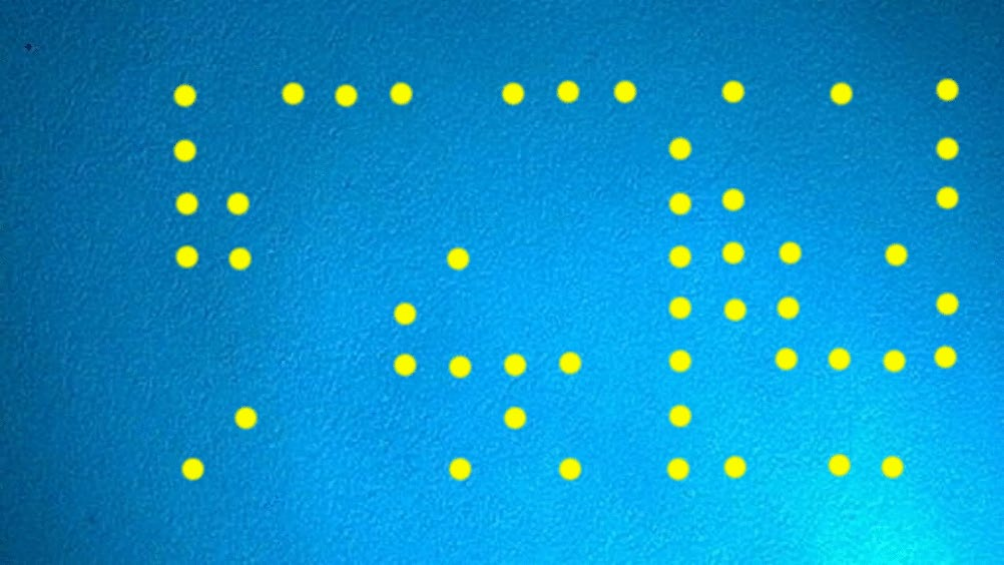
an example of a printed document with small, nearly invisible yellow dots that encode information. These dots are hard to see with the naked eye and require magnification to be noticeable.

Studies on printer identification (document source identification) focus on passive and active techniques. Passive techniques personalize printers by discovering built-in features in printed documents, aiming to discriminate printer models. This requires the creation of the printer’s instrument and improved analysis devices. Active techniques can regulate printer categories and brands based on features like banding frequencies, pattern noise, geometric distortion, printer profiles, and texture. Texture descriptors can be easily assessed over small regions and operate through changeable font type, font size, and printer age with high discrimination accuracy^3,4^. Active techniques in forensic science involve embedding an internal feature in a printed document, such as date and time of printing or printer serial number, to encode categorizing information. These features, which are yellow in color and appear unseen to the naked eye (see Fig. 1), are created by adjusting factors in printer machinery. However, this method is primarily used with color laser printers, which are limited in their use. Most printer identification schemes in forensic science use passive techniques, which change printing process factors to influence unpredicted printing quality. The intrinsic feature is connected to the printer’s electromechanical assets, making it difficult to counterfeit or eliminate. Yellow dots also hold encoded information that is barely visible to the naked eye^5–7^.

The printer identification task for Arabic manuscripts presents several challenges, including the possibility of multiple shapes for Arabic characters, various factors affecting performance like paper type, font size, printer consumable time, and font sizes, and the increasing complexity of falsifying documents with image processing tools. Additionally, some features of printed documents for printer identification may increase time and reduce cataloguing accuracy, as they may be redundant and non-informative. These factors make it a significant task to verify printed data effectively^8–10^. One critical component in document source identification task is the feature selection module. Once relevant features are selected, they are used to create a model that represents the unique characteristics of each printer. This model can then be used to compare and identify the source of unknown printed documents.

Feature modeling in printer forensics involves identifying, extracting, and analyzing various characteristics of printed documents to determine their source. The first step in feature modeling is to identify distinct characteristics that can be used to differentiate between printers. Features can be categorized into several types, such as geometric features, print quality defects, texture features, and microscopic features. Not all extracted features are equally useful for distinguishing between printers. Feature selection involves choosing the most relevant features that contribute significantly to the identification process. The selection of the most relevant features improves the performance of classification algorithms used to match a document to a specific printer. This leads to more accurate and reliable identification. With fewer but more relevant features, the computational load is reduced, leading to faster processing times. This is crucial when dealing with large datasets or when real-time analysis is required. Finally, by focusing on intrinsic features that are hard to alter, the system becomes more resilient to attempts at tampering or forgery^11–13^.

KNN classification offers several advantages when applied to document source identification applications, such as identifying the source printer of a document^14,15^. KNN is a lazy learner, meaning it does not involve a training phase. Instead, it stores all the training data and performs classification during the prediction phase. This can be advantageous when dealing with dynamic datasets that require frequent updates. It requires minimal assumptions about the data distribution, making it a great starting point for many classification problems. As well, KNN can be robust to noisy data. Since it relies on the majority voting among the neighbors, the influence of noise can be minimized if the majority of the nearest neighbors are correctly labeled. Besides, new data can be easily added to the existing dataset without the need for retraining, making KNN suitable for applications where new documents or sources are regularly introduced. Finally, KNN has only one key hyperparameter, the number of neighbors (k), simplifying the model selection process^6,13,14^.

The selection of the optimal value of K is a critical factor in the KNN algorithm, as it directly influences classification accuracy. In traditional KNN, this value is typically chosen through cross-validation or heuristic methods, often requiring extensive computational resources for large datasets. QKNN aims to enhance this process by utilizing quantum computing principles, allowing for faster and more efficient determination of the optimal *K*. By representing data as quantum states and using quantum algorithms to compute distances and perform nearest neighbor searches, QKNN can accelerate the selection of *K* while also reducing the computational complexity. Quantum algorithms are particularly beneficial in mitigating the curse of dimensionality, making them well-suited for high-dimensional feature spaces. Table 1 compares between traditional KNN and its Quantum version^15–18^.

**Table 1:** Comparative analysis of classification efficiency of KNN and QKNN machine learning algorithms.

Feature	KNN	QKNN
Data representation	Classical vectors	Quantum states
Distance calculation	Classical arithmetic	Quantum inner product, amplitude estimation
Computational efficiency	$O(N\cdot d)$ per query; where *N* is the number of data points and *d* is the dimensionality	$O(\sqrt{N})$ for certain distance calculations and neighbor searches
High dimensionality	Suffers from curse of dimensionality	Handles high-dimensional data effectively
Scalability	Limited	More scalable with large datasets
Training phase	None	None
Query phase	Classical distance calculation and search	Quantum state preparation and search
Noise and error resilience	Relatively robust	Sensitive to noise, needs error correction
Algorithm complexity	Simple and well-established	Complex, requires quantum computing knowledge
Real-time processing	Slower for large datasets	Faster due to quantum parallelism
Advanced feature extraction	Limited by classical methods	Enhanced by quantum feature mapping
Implementation	Easier with extensive libraries	Challenging, requires quantum programming

### Problem statement

Printer identification refers to the process of determining which specific printer was used to produce a given document. This can involve identifying the make, model, and sometimes even the individual printer that created a printed document based on various physical and digital characteristics. Accurately identifying the printer used to produce a document is crucial in various fields, including forensic investigations, security, and intellectual property protection. Traditional methods often rely on a limited set of features, which can result in ambiguous or inconclusive results. By incorporating a broader range of features and employing advanced feature modeling techniques, we can improve the accuracy and reliability of printer identification. Yet, high-dimensional data can lead to the curse of dimensionality, where the distance metrics become less meaningful as the number of dimensions increases. This can degrade the performance of the KNN classifier, making it difficult to distinguish between different printers effectively. KNN requires calculating the distance between the query point and all points in the training set. For large datasets with high-dimensional features, this can be computationally expensive.

### Motivation and contribution

Using QKNN classification for printer forensics offers a paradigm shift in the field, leveraging the principles of quantum mechanics to enhance accuracy and efficiency. Traditional KNN algorithms often rely on a predefined number of neighbors (*K*), limiting their adaptability to different types of data. In QKNN, the best *K* is estimated using a quantum search algorithm that efficiently explores the feature space. The quantum algorithm evaluates multiple potential values of *K* simultaneously, significantly reducing the search time compared to classical methods. Additionally, the accuracy of the QKNN classifier for each candidate *K* is used as a fitness function in a genetic algorithm. This allows the system to iteratively refine and select the optimal *K* value based on classification performance, ensuring that the best K is identified for the specific dataset and task. This paper explores a printer identification system for the Arabic alphabet, focusing on extracting GLCM from the printed letter ‘WaW’. The model uses bio-inspired feature selection to explore suitable feature subsets. The QKNN classifier is used for classification, with the 'leave one out’ technique used to compute the classification error rate. The accuracy of QKNN is measured as the fitness function for a genetic algorithm. The suggested system uses a quantum search algorithm to find the 'k' nearest neighbors. This step is significantly faster in a quantum context compared to classical algorithms.

The structure of this article is as follows: The relevant literature is presented in Sect. "State of the art". The suggested document source identification system’s design is presented in Sect. "Methodology". Results from experiments and comparisons to relevant literature and the suggested methodology are presented in Sect. "Results and discussion". The conclusion and plans for further research are summarized in Sect. "Conclusions".

### State of the art

The research and methodologies in document source identification can be categorized based on the features used for identification (physical, mechanical, and digital features), the techniques applied (image processing and computer vision, machine learning and artificial intelligence, and statistical and mathematical techniques), and the specific types of printers analyzed (Laser, Inkjet, and dot matrix printers)^19–21^. Convolutional Neural Networks (CNNs) of deep learning are used in Ref.^12^ to create a system that can automatically learn the features necessary to tackle the complex image categorization issue. Both systems have undergone systematic experiments. A feature-based Support Vector Machine (SVM) system outperforms a deep learning system for microscopic documents by a small margin. A new method for paper identification using hybrid features was suggested in Ref.^22^. This technique takes texture features from images that have been transformed using the GLCM method and feeds them into a CNN. The CNN then uses these features as inputs to extract yet another set of features. They had a 97.66% success rate when they used the recommended strategy to categorize seven of the most popular paper brands on the Korean market.

The authors in Ref.^23^ looked at how well Residual Network (ResNet) worked as a deep neural network architecture for a deep learning method to solve the printed document identification issue. ResNet can learn features that are very representative of the population and solve the vanishing gradient issue. In order to train their classification model, they used multiple ResNet variants—ResNet50, ResNet101, and ResNet152—on a large dataset of microprinted images that included microprinting patterns from different printers. To further improve the model’s performance and generalizability, the authors also used mix-up augmentation, a method that creates virtual training examples by interpolating image and label pairings.

The goal of printer-identification-based intelligent systems is to ascertain which printer is responsible for the creation of a given document. In certain cases, the majority of methods that rely on text dependent analysis could not be enough. To the best of the researchers’ knowledge in Ref.^19^, no research has been conducted on text-independent based on different word images generated from different kinds of laser printers. Consequently, they used the different grayscale word images to categorize the laser printer types. The collection includes four laser printer models with images that are forty thousand words long. A combination of the Local Binary Patterns (LBP) with KNN and the cubic SVM classifiers is used to categorize the various laser printer models. Similarly, the models for the laser printer are derived from the deep learning CNN model. They obtained high accuracy from KNN and cubic SVM classifiers of 97.2% and 97.9%, respectively, and 94.3% accuracy in the CNN model.

In Ref.^24^, the authors used a tiny dataset in addition to the whole article. Finding the document’s source printer without character, word, or patch segmentation and with tiny datasets is the goal of this paper’s three CNN-based approaches. To evaluate the proposed methods, we employ three distinct datasets consisting of 1,185, 1,200, and 2,385 documents, respectively. The first method relied on SVMs for classification and used thirteen pre-trained CNNs for feature extraction alone. The second method involves retraining an existing neural network to perform feature extraction and classification via transfer learning. Three, we can use CNNs built from scratch to extract features and SVMs to classify them. Many experiments were done with the three techniques, showing that the third technique gives the best result.

Recognizing printed papers in Chinese may be challenging due to the lack of unique characters in these documents, whereas typical techniques of printer source identification depend on specific characters to identify printed documents. To address this issue, this study in Ref.^7^ presented a text-independent printer source identification approach that models the printer’s timing connection using a graphical model and then extracts the text-independent printer’s timing characteristics. The approach has shown promising testing results and makes use of internal properties to enable recognition independent of particular characters. The results of the experiments demonstrate the practicality of the suggested strategy for document tracing.

A new method for predicting which printer model will generate a certain document is presented in Ref.^5^. A dataset of 41 inkjet printer models from popular manufacturers including HP, Canon, and Epson was created by collecting samples with a small number of letters written under diverse circumstances, including different printing modes, letter kinds, and fonts. Numerous methods were used to extract morphological characteristics from a set of microscopic images. With the remaining images serving as a training dataset, 30% were used for source printer prediction using discriminant analysis and the KNN technique. The printing circumstances were varied. In a validation examination, results showed that the accuracy was 98.6% when KNN and Quadratic Discriminant Analysis (QDA) were combined, compared to 96.3% when QDA was used alone. Forensic analysis of printed papers may so benefit from this method.

In Ref.^25^, a novel approach to identifying source color laser printers using the Counterfeit Protection System (CPS) pattern is detailed. A new Local Polar Pattern (LPP) for CPS pattern representation is first devised to provide scale and rotation invariance. A noise-robust CPS pattern extraction technique is then used, which is based on the LPP descriptor. On the other hand, a similarity measurement is established to determine how similar two CPS patterns are to one other. The last point is the introduction of a mechanism for identifying source color laser printers. In order to cut down on computation and data storage costs, the authors used the one-shot learning approach for CPS pattern recognition. Their technology outperforms deep neural networks and reaches state-of-the-art performance, according to the results of the experiment.

For the purpose of identifying the printer source of quick response codes, the authors of Ref.^26^ suggested a squeeze-excitation bottleneck residual network. In contrast to the bottleneck residual block’s few parameters, powerful feature extraction capability, and good expandability, the squeeze-excitation attention module focuses on features that represent the printer attributes, reduces the interference of useless information, and has low computational consumption. Thus, a small increase in parameters is all that is needed to effectively boost the performance. In comparison to existing approaches based on convolutional neural networks, the testing results show that the suggested method obtains a higher identification accuracy 98.77% when captured using a smartphone. The authors in Ref.^27^ aimed to minimize the amount of training features on several machine learning models for source printer identification while ensuring the excellent performance of the identifying results.

Based on their categorization as laser or other (inkjet or photocopier) machines, the current work in Ref.^28^ presented a unique method for determining the source of unknown printed documents using the first-ever application of Raman spectroscopy in conjunction with principal component analysis and partial least squares discriminant analysis. With a focus on the impact of printing direction, printing substrate, and printing technology, the authors of Ref.^29^ offered a statistical study of the printing patterns at a microscopic scale. Using the shape descriptor indexes, the printing materials and technologies can be distinguished on a tiny scale, despite the research showing that printing direction has a little impact. Consequently, methods for printing source identification are created using support vector machines and random forests, using shape descriptor indexes as features. With intricate geometric-shape patterns, both algorithms reach an impressive 92% classification accuracy rate.

A printer-specific pooling descriptor that enhances the performance of a printer-specific local texture descriptor on two datasets was thoroughly analyzed and suggested in Ref.^30^. Instead of a complicated machine learning-based classifier, the suggested pooling works well in cross-font situations due to a simple correlation-based prediction method. Printer hardware causes subtle, invisible distortions to the positions and forms of printed characters throughout the printing process. Geometric distortions are the name given to these alterations. Each printer often has their own distinct profile, sometimes called a signature, which may be used to categorize printers. A new method for determining a document’s source is presented in Ref.^31^, which also suggested a set of characteristics for describing geometric distortions at the text line level. The system outperforms the state-of-the-art geometric distortion system and provides much better accuracy with a minor training size limitation, according to extensive trials conducted on a set of fourteen printers.

The usefulness of CNN-learned deep visual features in the characterization of the printer source was examined in Ref.^32^. For the purpose of extracting features, images of printed documents are split into tiny patches and characters. Researchers may conduct their studies in either a text-dependent or text-independent mode thanks to the integrated off-the-shelf recognition engine. Conducting experiments on a typical dataset consisting of papers from 20 different printers, we find that patches achieve an identification rate of 95.52% while characters achieve an identification rate of 98.06%. Complete images of printed documents may be used by the suggested method, which achieves high identification rates, in contrast to many current methods that depend on pre-segmented characters and provide results by comparing the same characters only.

The research in Ref.^33^ looked at how well the Auto-Machine Learning-Based Method (AutoML) could analyze microscopic images of printed documents in order to identify the printer that produced them. Three candidates from well-known ML models and two from AutoML are nominated for a competition in this work. The experiments demonstrated the limitations of conventional approaches and the benefits of using AutoML. In this case, the authors want to highlight the effectiveness of ensemble techniques in order to get the most optimal model for their experimental dataset. Additionally, it is acknowledged that AutoML may be trained to handle various degrees of ambiguity in printed patterns.

An approach for identifying the source printer and classifying the questioned document into one of the printer classes was presented in Ref.^34^. The identification results were significant for a dataset of 1200 papers from 20 different printers, including 13 laser and 7 inkjet printers. Their technique detects printers by combining global features such as the Histogram of Oriented Gradient (HOG) with local features such as LBP descriptors. SVMs, aggregate bootstrapping (bagging), Decision Trees (DTs), KNNs, and Random Forests (RFs) are some of the classifiers that have been used for classification. A 96% success rate was achieved by the adaptive boosting classifier.

Based on cascaded learning of deep neural networks, the authors in Ref.^35^ described a solution for color laser printer identification. Prior to applying halftone color decomposition to the synthetic dataset, the refiner network is trained using adversarial training. After that, the ConvNet that stands for CNN, which decomposes halftone colors is trained using the updated dataset. The printer’s identifying ConvNet receives the taught information about halftone color decomposition, which improves the accuracy of identification. Halftone images created by candidate source printers are used to train the printer detecting ConvNet. Unlike other approaches, this one takes rotation and scaling robustness into account during training. The suggested technique significantly outperforms the current source color laser printer identification methods, according on the testing results.

### The need to extend the related work

Most existing research in printer forensics has focused on classical machine learning methods and physical analysis techniques that examine the tangible characteristics of printed documents to identify unique signatures and artifacts left by specific printers. Studies have demonstrated the feasibility of identifying printer-specific signatures, but challenges remain in terms of accuracy, computational efficiency, and handling large datasets. While quantum computing holds promise, its application in printer forensics is still in its infancy. Extending related work, for the first time, to include quantum approaches, QKNN in our case, can significantly enhance the field. Furthermore, investigating optimal methods for feature extraction and quantum encoding specific to printer forensics will help in maximizing the benefits of quantum classifiers. By extracting optimal physical features from printed documents and leveraging the computational power of quantum computing, printer forensic investigators can achieve more reliable and rapid results. This hybrid approach not only improves the precision of printer forensic analyses but also provides a scalable solution for handling large printer forensic datasets.

While quantum computing has led to the development of quantum classifiers, a review of Google Scholar reveals that no research has yet explored their application in printer forensics. This study aims to investigate the impact of QKNN on determining the optimal value of K based on the characteristics of the dataset, and to evaluate its effect on the accuracy of document source identification. By leveraging quantum algorithms, QKNN has the potential to enhance the performance and precision of classification models, offering a more efficient and accurate approach to identifying the source of printed documents. This research seeks to demonstrate how quantum methods can improve upon traditional KNN techniques in this domain.

## Methodology

The framework of the printer identification system that is based on image texture analysis is described in this section. The GLCM approach is used by the model in order to get the features of a certain printer. Following this, the Genetic Algorithm (GA) method is adapted in order to choose the feature set that is competent enough to be utilized for classification. In-depth explanations will be provided for each stage. The dataset was gathered from ten distinct printers, each with a unique model and serial number, from various brands. The high-resolution image seems brittle, and its texture will typically be more flawless and colorful, therefore documents are scanned at 1200 dpi with 8 bits per pixel (greyscale) after data collection. Then, from the isolated character “و” all of the features have been extracted.

"The 'و' character (pronounced ‘waw’) is often used in Arabic and Persian to indicate 'and,' serving as a conjunction to connect words and phrases. However, if we’re referring to its use specifically in printer identification, it might be due to its uniqueness in shape, which makes it easily distinguishable. The character "و" has several unique shape characteristics that make it particularly suitable for printer identification: (1) Curved Shape: The character "و" consists of a single, smooth, curved stroke, which is easily recognizable. (2) Lack of Diacritics: Unlike many other Arabic characters, "و" does not have diacritical marks (such as dots or accents), reducing the chance of misreading or misprinting. (3) Vertical Alignment: the character "و" is vertically aligned and does not have any horizontal extensions, making it less likely to blend with surrounding text and easier to spot. (4) Consistency in Form: The shape of "و" remains consistent regardless of its position in a word (beginning, middle, or end), unlike some other Arabic characters that change shape depending on their position. (5) Minimalistic Design: The minimalistic and clean design of "و" ensures it remains legible and distinguishable even at small sizes or low resolutions, which is crucial for printer identification.

### Image pre-processing stage

**Fig. 2:**
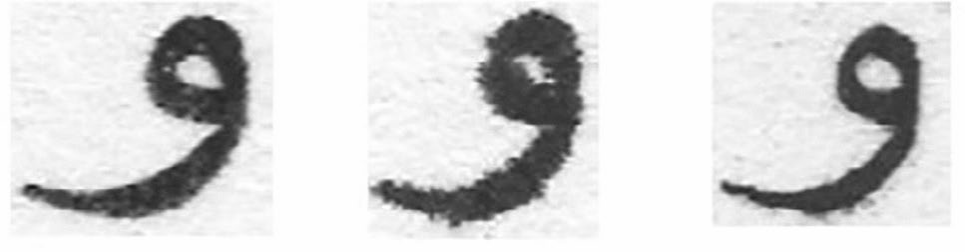
Character "و" printed from different printers.

Printers from different brands vary in how they handle printing Arabic characters due to differences in font rendering, print resolution, and character handling algorithms. Arabic script, which features connected letters, complex ligatures, and right-to-left alignment, requires precise rendering for clarity and accuracy. For instance, printers with lower resolution may struggle to produce the fine details of Arabic calligraphy, leading to jagged or broken characters, especially in curved strokes. In contrast, higher-end printers or brands with better support for complex fonts (e.g., HP or Canon) tend to manage these complexities better, producing smoother, more legible Arabic text. Additionally, different brands might employ unique software or drivers to optimize font spacing, line thickness, or ligature handling. For example, Brother Printers might apply less smoothing to Arabic script, while Epson models could emphasize higher contrast for improved readability. Such differences affect the overall quality and consistency of printed Arabic characters across various printers^1–3^. Figure 2 shows the impact of printer brand variability on Arabic character rendering “و”.

**Fig. 3:**
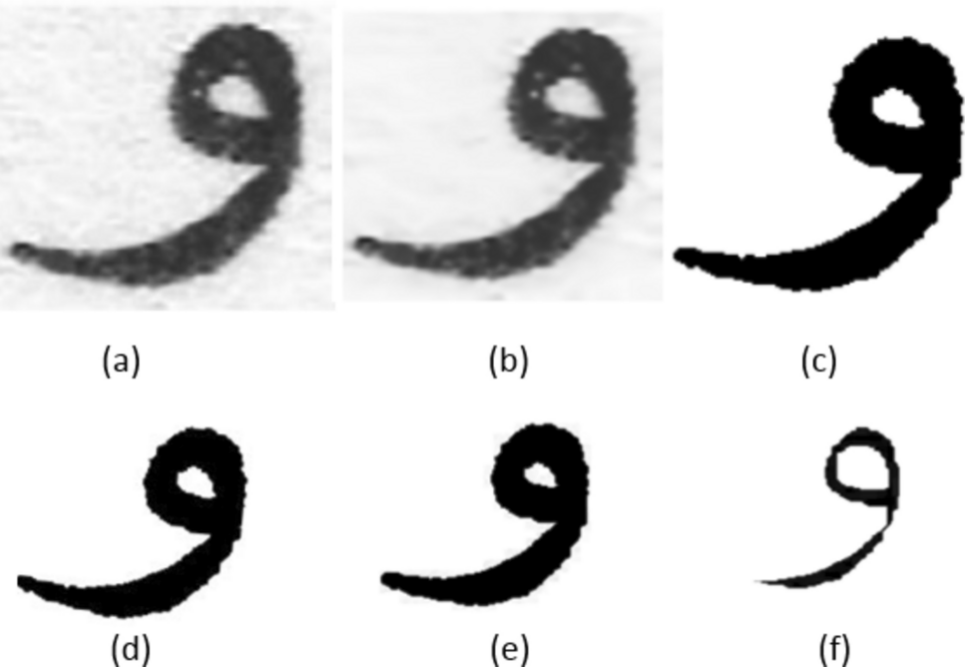
"Pre-processing techniques for enhancing printer identification of the Arabic character “و”. (**a**) Original scanned image, (**b**) Noise reduction, (**c**) Binarization; (**d**) Resize and orientation, (**e**) Dilation 3 × 3; (**f**) Erosion 5 × 5.

Pre-processing an image of the character "و" for printer identification offers several benefits, enhancing the accuracy and efficiency of the identification process, which includes: (1) Noise Reduction: Removes unwanted noise from the image, ensuring that the character is accurately represented. (2) Enhancement of Features: Enhances the character’s distinct features, making it easier for the recognition system to identify the character correctly. (3) Standardization: Normalizes the size and orientation of the character, providing a consistent input for the identification system. (4) Uniform Background: Eliminates variations in background that could interfere with character recognition. (5) Improved Legibility: Smoothing and morphological operations can enhance the legibility of character in low-quality or low-resolution images^36^. In our case, conversion to grayscale, binarization, noise reduction, image cropping, and thinning operations are implemented to make images of the character "و" suitable for further analysis or classification tasks. Figure 3 visualizes some sample images for the reader’s understanding before and throughout the pre-processing stage.

### Feature extraction based on GLCM

**Fig. 4:**
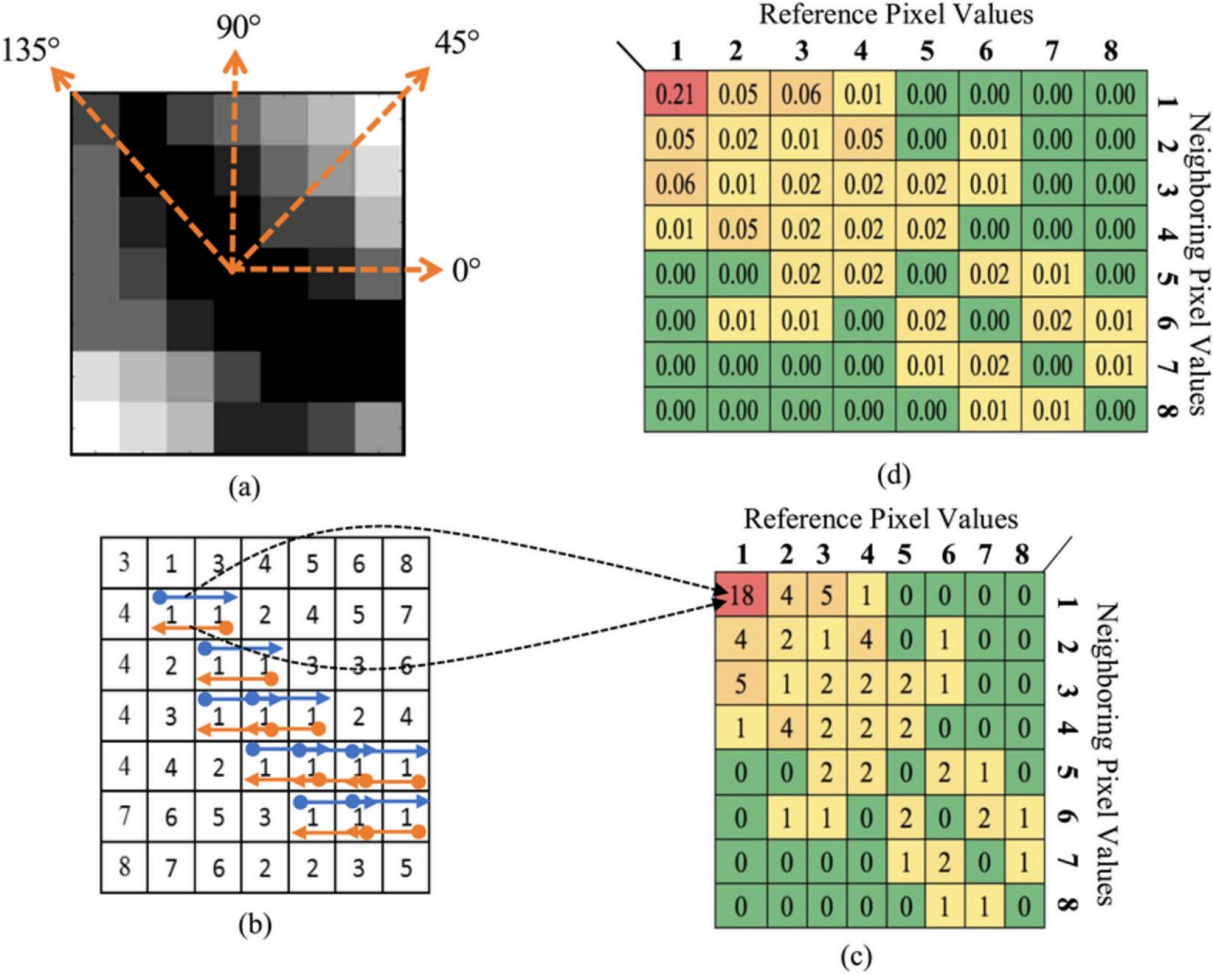
An example of calculating a GLCM with 1 pixel separation, 0° direction, and 1–8 intensity levels. (**a**) Sample grayscale image. Equivalent gray-level intensities range from 1 to 8 bits are shown in (**b**). The arrows show the number of pixel pair occurrences (1, 1) for the selected direction and pixel separation. (**c**) The input image (**a**)'s corresponding GLCM count matrix, which shows the frequency of reference and surrounding pixel occurrence. (**d**) Corresponding GLCM probability matrix.

The GLCM is a powerful tool for texture analysis, which can be highly beneficial in printer identification applications. Different printers produce unique textural patterns due to variations in printing mechanisms, toner/ink quality, and print resolution. GLCM helps in capturing these unique signatures^37^. GLCM features are less sensitive to noise compared to pixel intensity-based features. This robustness is crucial for dealing with real-world documents that might have scanning noise or degradation. GLCM can generate multiple texture features, such as contrast, correlation, energy, homogeneity, and entropy, providing a comprehensive set of attributes for classification^37,38^. These measures can be highly discriminative for printer identification, helping to distinguish between minute variations in print textures. By calculating GLCMs at different orientations and distances, it captures directional and scale-dependent textural information, adding richness to the feature set (see Fig. 4). Each combination of distance and angle produces a separate GLCM. Therefore, if we have *N* distances and *M* angles, we will have *N*×*M* GLCMs^39^. Table 2 shows a comparative analysis between GLCMs and other common texture analysis techniques for printer identification. In printer identification, GLCMs stand out due to their ability to capture detailed texture information and directional sensitivity, combined with robustness to noise and computational efficiency.

**Table 2:** Comparative analysis between GLCMs and other common texture analysis techniques for printer identification.

Criteria	GLCM	LBP	Wavelet transform (WT)	Fourier transform (FT)
Texture information	Captures spatial relationships between pixels, providing detailed texture information	Captures local texture information, often used for recognizing simple textures	Captures both frequency and spatial information, providing multi-resolution analysis	Captures frequency information, useful for identifying periodic textures
Directional sensitivity	Analyzes textures in multiple directions (0°, 45°, 90°, 135°)	Not inherently directional but can be adapted for directional analysis	Can analyze textures at different scales and orientations	Primarily analyzes frequency components, less focus on spatial orientation
Noise robustness	Generally robust to noise due to averaging effects of co-occurrence matrix	Sensitive to noise; small changes can significantly affect LBP patterns	Robust to noise due to multi-resolution decomposition	Sensitive to noise; high-frequency components can be significantly affected
Discriminative power	High; provides multiple statistical measures (contrast, correlation, energy, homogeneity)	Moderate; primarily captures local texture information	High; captures both coarse and fine texture details	Moderate; primarily captures global texture information
Computational efficiency	Relatively efficient to compute; straightforward implementation	Very efficient to compute; simple implementation	More computationally intensive due to multi-scale analysis	Efficient for computing frequency components, but can be complex for large images
Implementation complexity	Simple; well-documented and easy to implement	Very simple; easy to implement and use	Complex; requires more sophisticated algorithms and parameter tuning	Moderate; FT is straightforward, but interpreting results can be complex
Empirical success	Proven effectiveness in printer identification and other texture-based applications	Effective for simple texture recognition tasks, less so for complex textures	Proven for detailed texture analysis, used in many image processing applications	Effective for identifying periodic textures, less effective for non-periodic textures
Hybrid compatibility	Easily combined with other techniques for enhanced performance	Can be combined with other methods, especially for enhancing local texture analysis	Often combined with other techniques for comprehensive texture analysis	Can be used with spatial domain techniques for a hybrid approach
Applications	Widely used in texture-based classification, including printer identification	Used in texture classification, face recognition, and medical imaging	Used in image compression, texture analysis, and signal processing	Used in image processing, signal analysis, and pattern recognition

In a document source identification system for printer forensics, the optimal values for the “*M*” (angles) and “*N*” (distances) parameters of the GLCM for feature extraction are critical in capturing the texture patterns and noise introduced by different printers. The values we choose for these parameters should balance between capturing meaningful texture variations and maintaining computational efficiency. To balance meaningful texture analysis and computational efficiency, four angles—0° (horizontal), 45° (diagonal), 90° (vertical), and 135° (anti-diagonal)—are typically used, as printer noise patterns may vary across these directions due to mechanical factors. This multi-directional approach enhances the robustness of feature extraction by capturing noise from multiple orientations. For the *N* (distances) parameter, a small value of 1 to 2 pixels is ideal (*N* = 2 in our case), especially for high-resolution images (e.g., 1200 dpi), as it captures the fine-grained texture variations and subtle noise patterns that are crucial for distinguishing between printers^37^.

GLCM is applied for feature extraction in this study because it excels at capturing spatial texture relationships, which are crucial for distinguishing subtle noise and texture variations introduced by different printers. Compared to other approaches like Histogram of Oriented Gradients (HOG) and LBP, which focus more on edge and shape features, GLCM provides a more detailed analysis of texture, essential for printer forensics where such fine patterns are key identifiers. Additionally, GLCM is more robust to noise, which is often present in printed documents, and offers greater interpretability than deep learning-based features, which, while powerful, often function as “black box” models with less transparency. GLCM is also computationally efficient, requiring less processing power than deep learning methods, making it suitable for real-time or resource-limited environments. Overall, GLCM’s ability to capture detailed, noise-resistant texture features with relatively low computational cost makes it a strong choice for printer identification tasks in this study^38^.

### Feature selection using GA

**Fig. 5:**
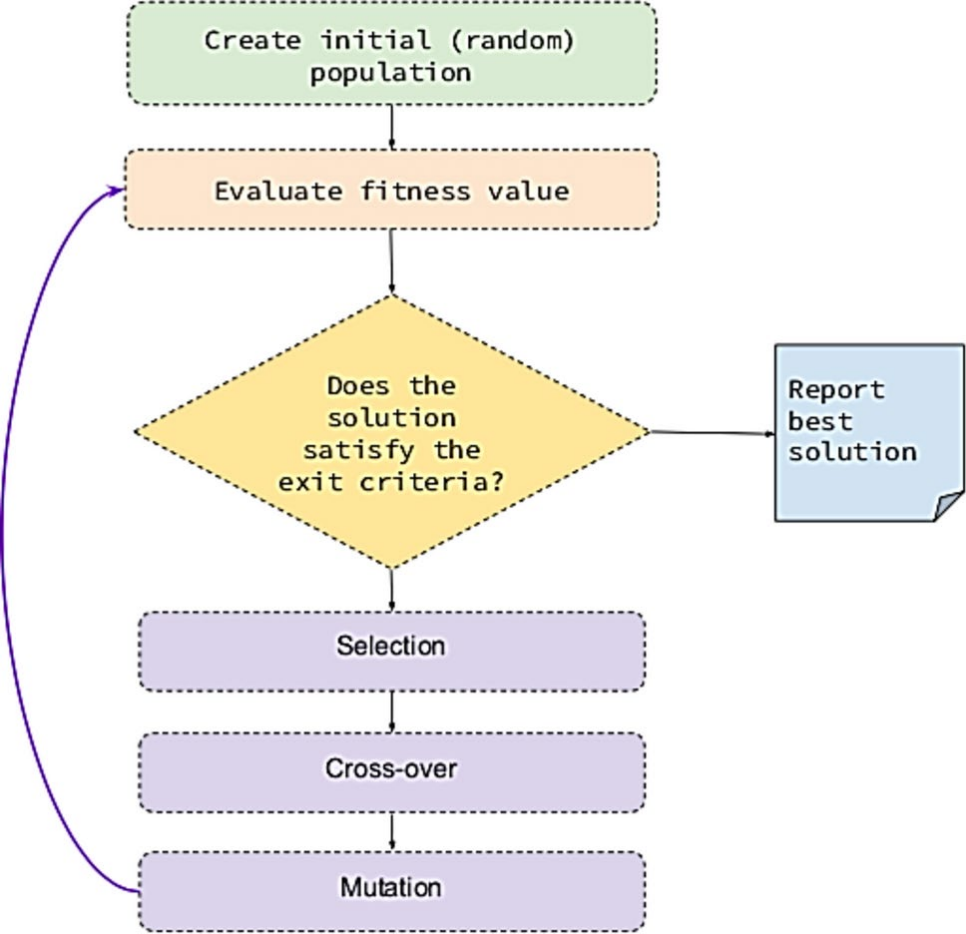
Schematic representation of the GA procedure.

In this work, we extracted 22 descriptors from the printed documents of the printer dataset. The printer dataset covers 1,000 printed documents (images) of 10 kinds of printers. Using a GA for feature selection in printer identification offers several benefits, particularly due to its ability to handle complex, high-dimensional search spaces and find optimal or near-optimal solutions efficiently. Printer identification may involve a large number of features extracted from various image processing techniques, including GLCM. GAs are well-suited for exploring high-dimensional feature spaces. GAs can effectively reduce the dimensionality of the feature set by selecting the most relevant features, thereby improving computational efficiency and model performance^40^. By selecting the most relevant features, GAs can improve the accuracy of the printer identification model. Irrelevant or redundant features are excluded, leading to better generalization on unseen data. In general, with fewer, more relevant features, the model is less likely to overfit to the training data, resulting in improved performance on validation and test sets. Figure 5 illustrates the main steps of GA procedure^41^. In our case, the fitness function of each population’s chromosome is calculated via QKNN-based classification error and the cardinality of the nominated features (descriptors). The main objective is to achieve the balance between the classification error minimization with a minimum set of descriptors.

GA is preferred for feature selection in this study due to its ability to explore a vast search space efficiently and handle complex, non-linear interactions between features. Unlike filter methods, which evaluate features independently of the model, GA takes into account the feature set’s collective contribution to classification performance. Compared to wrapper methods, which can be computationally expensive as they evaluate each possible feature subset, GA offers a more efficient heuristic approach by simulating natural selection, iteratively evolving feature sets to find optimal or near-optimal solutions. Embedded methods, while integrated into model training, may be limited by the specific model used, whereas GA is model-agnostic and can be applied flexibly across various classifiers. Its adaptability and capacity to balance exploration and exploitation make GA particularly effective for identifying the most relevant features in complex tasks like printer forensics, where subtle feature interactions are key to accurate identification^40,41^.

In a document source identification system for printer forensics, GA are often used for optimizing feature selection or model parameters. The optimal values for the GA parameters—population size, number of generations, crossover probability, and mutation probability—should strike a balance between exploration and exploitation of the search space, as well as computational efficiency. The optimal parameters for a GA are typically as follows: a population size of 50 to 100 individuals is ideal, as it provides sufficient diversity without significantly increasing computational cost. Running the GA for 100 to 200 generations allows for thorough exploration of the search space, capturing subtle noise patterns and texture differences in printed documents. A crossover probability of 0.7 to 0.9 ensures effective combination of solutions, speeding up convergence by exploring a wide range of feature interactions. Meanwhile, a mutation probability of 0.01 to 0.05 introduces enough randomness to prevent premature convergence, maintaining diversity without destabilizing the optimization process. These values offer a balanced approach to achieving accurate printer identification while keeping computational demands manageable^40,41^.

In our implementation, the GA is set up with the following detailed configuration: a population size of 100 individuals, which determines the number of candidate solutions in each generation. The algorithm is run for 100 generations, allowing the population to evolve over multiple iterations. A crossover probability of 0.9 is applied, meaning that 90% of the individuals in the population undergo crossover to exchange genetic information, fostering the creation of new, potentially superior offspring. Additionally, a mutation probability of 0.01 is used, introducing small random changes in 1% of the population to maintain genetic diversity and prevent premature convergence.

### QKNN classification algorithm

Using a QKNN classifier for printer identification applications can be justified by several potential advantages that quantum computing offers over classical computing^15,18^. Quantum computing introduces new ways to measure distances in feature space, possibly leading to more accurate identification. Quantum states can represent data in higher-dimensional spaces, which might capture more intricate details of printer characteristics. In this work, we utilized a QKNN’ algorithm in which the calculation of the Euclidean distances is based on a novel quantum encoding with low qubit requirements and a simple quantum circuit, making the implementation particularly advantageous^42^.

Let us consider a training set $\mathcal{U}=\left\{{{\varvec{u}}}_{0},\dots \dots {{\varvec{u}}}_{{\varvec{N}}-1}\right\}$ of real valued data instances ${{\varvec{u}}}_{{\varvec{j}}}\in {\mathbb{R}}^{{\varvec{d}}}$ , and let $\mathcal{L}=\left\{{{\varvec{l}}}_{0},\dots \dots {{\varvec{l}}}_{{\varvec{N}}-1}\right\}$ be the set of corresponding labels. In addition, let us consider a test instance ${{\varvec{u}}}^{\boldsymbol{^{\prime}}}\in {\mathbb{R}}^{{\varvec{d}}}$ , whose label is unknown. The preprocessing step of the algorithm consists in centering and normalizing the data features into the range $\left[-\frac{1}{2\sqrt{{\varvec{d}}}},\frac{1}{2\sqrt{{\varvec{d}}}}\right]$. In this way, the maximum norm of the resulting vectors turns out to be 1/2 and the maximum (squared) Euclidean distance turns out to be 1^42^. Let $\mathcal{V}=\left\{{{\varvec{v}}}_{0},\dots \dots {{\varvec{v}}}_{{\varvec{N}}-1}\right\}$ and ${{\varvec{v}}}^{\boldsymbol{^{\prime}}}$ be the training set and the test instance after the preprocessing step. The quantum circuit is then initialized in the state

1 $$|\boldsymbol{ }{\varvec{\psi}}\rangle =|0\rangle \otimes \left(\frac{1}{\sqrt{2}}\boldsymbol{ }\boldsymbol{ }\left(|0\rangle \left|\boldsymbol{\alpha }\rangle +|1\rangle \right|{\varvec{\upbeta}}\rangle \right)\right),$$

2 $$|\alpha \rangle =\frac{1}{\sqrt{N}}\sum_{j=0}^{N-1}|j\rangle \sum_{i=0}^{F-1}{x}_{ji}|i\rangle ,$$

3 $$|\beta \rangle =\frac{1}{\sqrt{N}}\sum_{j=0}^{N-1}|j\rangle \sum_{i=0}^{F-1}{{x}^{\prime}}_{ji}|i\rangle .$$

*F* is a positive integer value depending on the encoding used, while ${{\varvec{x}}}_{{\varvec{j}}}={\left\{{{\varvec{x}}}_{{\varvec{j}}{\varvec{i}}}\right\}}_{{\varvec{i}}=0,\dots \dots ,{\varvec{F}}-1}$ and ${{{\varvec{x}}}^{\boldsymbol{^{\prime}}}}_{{\varvec{j}}}={\left\{{{{\varvec{x}}}^{\boldsymbol{^{\prime}}}}_{{\varvec{j}}{\varvec{i}}}\right\}}_{{\varvec{i}}=0,\dots \dots ,{\varvec{F}}-1}$ represent the quantum encoded versions of the preprocessed training and test data, respectively. Therefore, the number of qubits required is $2+\lceil{{\varvec{l}}{\varvec{o}}{\varvec{g}}}_{2}{\varvec{N}}\rceil+\lceil{{\varvec{l}}{\varvec{o}}{\varvec{g}}}_{2}{\varvec{F}}\rceil$. In this case, encoding is defined as follows in which ${{\varvec{v}}}_{{\varvec{j}}{\varvec{i}}}$ being the i-th feature of the j-th preprocessed training instance, and ${{{\varvec{v}}}^{\boldsymbol{^{\prime}}}}_{{\varvec{i}}}$ being the i-th feature of the preprocessed test instance.

4 $${x}_{ij}=\left\{\begin{array}{c}\begin{array}{c} \begin{array}{cc}\frac{2}{\sqrt{3}} {v}_{ji}& 0\le i<d\end{array}\\ \begin{array}{cc}\frac{2}{\sqrt{3}}{v}_{j\left(i-d\right)}& d\le i<2d\end{array}\\ \begin{array}{cc}\frac{2}{\sqrt{3}}\Vert {v}_{j}\Vert & i=2d\end{array}\\ \begin{array}{cc}0& i=2d+1\end{array}\end{array}\\ \begin{array}{cc}\sqrt{1-4{\Vert {v}_{j}\Vert }^{2}} & i=2d+2\end{array}\end{array}\right.$$

5 $${{{\varvec{x}}}^{\boldsymbol{^{\prime}}}}_{{\varvec{j}}{\varvec{i}}}=\left\{\begin{array}{c}-\frac{2}{\sqrt{3}} {{{\varvec{v}}}^{\boldsymbol{^{\prime}}}}_{{\varvec{i}}\boldsymbol{ }\boldsymbol{ }\boldsymbol{ }\boldsymbol{ }\boldsymbol{ }} 0\le i<d\\ -\frac{2}{\sqrt{3}} {{{\varvec{v}}}^{\boldsymbol{^{\prime}}}}_{\left({\varvec{i}}-{\varvec{d}}\right)\boldsymbol{ }\boldsymbol{ }} d\le i<2d\\ \frac{2}{\sqrt{3}}\Vert {{\varvec{v}}}_{{\varvec{j}}}\Vert i=2d \\ \sqrt{1-\frac{4}{3}\left(2\Vert {\varvec{v}}{\boldsymbol{^{\prime}}\Vert }^{2}+{\Vert {{\varvec{v}}}_{{\varvec{j}}}\Vert }^{2}\right)\boldsymbol{ }} i=2d+1\\ 0 i=2d+2,\end{array}\right.$$

**Fig. 6:**
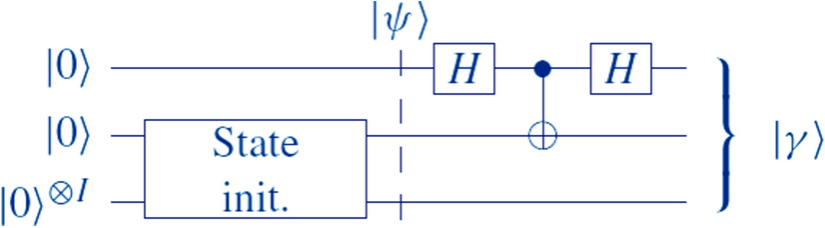
The corresponding quantum circuit of Bell-H operation.

Bell State Hadamard (Bell-H) is performed using Qiskit’s simulation toolbox to implement the QKNN classification algorithm on classical computers^43^. It consists of a Hadamard gate applied to the first qubit, a Controlled NOT (CNOT) gate with the first qubit as control and the second qubit as target, and another Hadamard gate applied to the first qubit (see Fig. 6). The output state obtained after the Bell-H operation is:

6 $$y\rangle =\frac{1}{2}\left(|0\rangle \otimes \left(\frac{1}{\sqrt{2}} \left|0\rangle \right|\alpha \rangle +\left|0\rangle \right|\beta \rangle -\left|1\rangle \right|\beta \rangle \left|1\rangle \right|\alpha \rangle \right)\right)+ \left|1\rangle \otimes \left(\frac{1}{\sqrt{2}} \left|0\rangle \right|\alpha \rangle -\left|0\rangle \right|\beta \rangle +\left|1\rangle \right|\beta \rangle -\left|1\rangle \right|\alpha \rangle \right)\right),$$

7 $${\varvec{F}}{\varvec{i}}{\varvec{n}}{\varvec{a}}{\varvec{l}}\boldsymbol{ }{\varvec{s}}{\varvec{t}}{\varvec{a}}{\varvec{t}}{\varvec{e}}=\boldsymbol{ }|{\varvec{\delta}}\rangle =\frac{1}{\sqrt{{\varvec{N}}}}\boldsymbol{ }\sum_{{\varvec{j}}=0}^{{\varvec{N}}-1}[\sqrt{1-{\varvec{s}}\left({{\varvec{v}}}_{{\varvec{j}}},{{\varvec{v}}}^{\boldsymbol{^{\prime}}}\right)}|0\rangle +\sqrt{{\varvec{s}}\left({{\varvec{v}}}_{{\varvec{j}}},{{\varvec{v}}}^{\boldsymbol{^{\prime}}}\right)}\boldsymbol{ }\left|1\rangle \right]|{\varvec{j}}\rangle ,$$

${\varvec{s}}\left({{\varvec{v}}}_{{\varvec{j}}},{{\varvec{v}}}^{\boldsymbol{^{\prime}}}\right)$ is the similarity measure related to the squared Euclidean distance between ${{\varvec{v}}}_{{\varvec{j}}}$ and ${{\varvec{v}}}^{\boldsymbol{^{\prime}}}$; hence, the lower the distance, the higher the ${\varvec{s}}\left({{\varvec{v}}}_{{\varvec{j}}},{{\varvec{v}}}^{\boldsymbol{^{\prime}}}\right)$ value. Specifically, ${\varvec{s}}\left({{\varvec{v}}}_{{\varvec{j}}},{{\varvec{v}}}^{\boldsymbol{^{\prime}}}\right)$ is given by

8 $${\varvec{s}}\left({{\varvec{v}}}_{{\varvec{j}}},{{\varvec{v}}}^{\boldsymbol{^{\prime}}}\right)={\varvec{P}}\left({{\varvec{q}}{\varvec{u}}{\varvec{b}}{\varvec{i}}{\varvec{t}}}_{1}=1|{\varvec{j}}\right)=\frac{1}{2}\left(1-\langle {{\varvec{x}}}_{{\varvec{j}}},{{\varvec{x}}}_{{\varvec{j}}}^{\boldsymbol{^{\prime}}}\rangle \right),$$

9 $$\langle {{\varvec{x}}}_{{\varvec{j}}},{{\varvec{x}}}_{{\varvec{j}}}^{\boldsymbol{^{\prime}}}\rangle =\frac{4}{3}\left({\Vert {{\varvec{v}}}_{{\varvec{j}}}\Vert }^{2}-2\langle {{\varvec{v}}}_{{\varvec{j}}},{{\varvec{v}}}^{\boldsymbol{^{\prime}}}\rangle \right)$$

Quantum Bit (${{\varvec{q}}{\varvec{u}}{\varvec{b}}{\varvec{i}}{\varvec{t}}}_{1})$ is the first qubit in the circuit, and $\langle {{\varvec{x}}}_{{\varvec{j}}},{{\varvec{x}}}_{{\varvec{j}}}^{\boldsymbol{^{\prime}}}\rangle$ is strictly related to the squared Euclidean distance between ${{\varvec{v}}}_{{\varvec{j}}}$ and ${{\varvec{v}}}^{\boldsymbol{^{\prime}}}$. By iterating the circuit execution and the measurement process, the joint probabilities ${\varvec{P}}\left(0,{\varvec{j}}\right)$ and ${\varvec{P}}\left(1,{\varvec{j}}\right)$ are estimated as relative frequencies, allowing in turn the estimation of the Euclidean distances. Indeed, the following relationships hold:

10 $$P\left(0,j\right)=\frac{1+\langle {x}_{j},{x}_{j}^{\prime}\rangle }{2N} \Rightarrow \langle {x}_{j},{x}_{j}^{\prime}\rangle =2N*P\left(0,j\right)-1,$$

11 $$P\left(1,j\right)=\frac{1-\langle {x}_{j},{x}_{j}^{\prime}\rangle }{2N} \Rightarrow \langle {x}_{j},{x}_{j}^{\prime}\rangle =1-2N*P\left(1,j\right),$$

12 $${\varvec{E}}{\varvec{u}}{\varvec{c}}{\varvec{l}}{\varvec{i}}{\varvec{d}}{\varvec{e}}{\varvec{a}}{\varvec{n}}\boldsymbol{ }{\varvec{D}}{\varvec{i}}{\varvec{s}}{\varvec{t}}{\varvec{a}}{\varvec{n}}{\varvec{c}}{\varvec{e}}={\varvec{d}}\left({{\varvec{v}}}_{{\varvec{j}}},{{\varvec{v}}}^{\boldsymbol{^{\prime}}}\right)=\sqrt{\frac{3}{4}\langle {{\varvec{x}}}_{{\varvec{j}}},{{\varvec{x}}}_{{\varvec{j}}}^{\boldsymbol{^{\prime}}}\rangle +\Vert {{\varvec{v}}}^{\boldsymbol{^{\prime}}}{||}^{2}}$$

Once all the Euclidean distances ${\varvec{d}}\left({{\varvec{v}}}_{{\varvec{j}}},{{\varvec{v}}}^{\boldsymbol{^{\prime}}}\right)$ have been estimated, the training elements are classically sorted according to them. Then, the KNN are identified, and the test instance is classified by means of a majority voting on the labels of the nearest neighbors.

The optimal *K* value for QKNN classifier varies with sample size: for a sample size of 1,000, the ideal K is between 3 and 5, as this range enhances sensitivity to local patterns and subtle differences in printer features, crucial for accurate classification. This smaller K also helps manage the bias-variance tradeoff, allowing the classifier to make informed decisions based on its closest neighbors. In contrast, for a sample size of 10,000, the optimal K increases to 5–10, providing more stability and improved generalization by averaging out noise and reducing the influence of outliers. This balance allows the QKNN classifier to effectively utilize available data and adapt to variations in sample size^42^. Additionally, the fitness function in a genetic algorithm refines the selection of K based on accuracy, allowing the classifier to capture sufficient neighborhood information without being overly sensitive to noise. This approach optimizes K based on dataset-specific features, balancing both accuracy and computational efficiency.

In our implementation, the *K* parameter is configured with specific values based on the sample size. For smaller datasets, where the sample size is limited, K is set to 3, ensuring that the model maintains enough neighbors to make reliable predictions while minimizing the impact of noise. For larger datasets, K is increased to 5, allowing the model to account for more neighbors and leverage the additional data points for greater accuracy. This adaptive approach ensures that the parameter is appropriately tuned to the dataset size, balancing precision and generalization across different sample sizes.

To conclude, by applying QKNN to printer forensics for the first time, this method enhances the ability to detect and identify subtle differences in printed documents, offering a faster and more accurate solution than traditional KNN algorithms. This combination of quantum computing and forensic analysis marks a fundamental shift in how such identification tasks can be approached, providing the field with a cutting-edge tool that surpasses existing methodologies.

## Results and discussion

Google Colab Python version 2.7 was used to implement the system prototype in a modular manner with the objective of verifying the validity of the proposed document source identification for printer recognition. The system was tested on a DellTM InspironTM N5110 laptop, Dell Computer Corporation, Texas, which included the following specifications: 64-bit Windows 7 Home Premium, 4.00 GB RAM, Intel (R) Core (TM) i5-2410 M CPU, 2.30 GHz. Table 3 displays a variety of models and serial numbers for ten printers from a variety of manufacturers that are frequently employed in the digital evidence laboratory.

**Table 3:** A variety of models and serial numbers for ten printers.

	Brand	Model No.	Serial No.
P1	NashuaTec	Spc410Aficio	Q7088600729
P2	Hp	Laser1102	Vnc4841849
P3	Samsung	M332x	Zdfbbjag30006mw
P4	Samsung	M332x	Zdfbbjcg300023e
P5	Hp	LaserJet1018	Cncig74912
P6	Canon	LBP3010B	MXBA909688
P7	Samsung	M332x	Zdfbbjag300008e
P8	HP	Laser1100	CED96852
P9	Canon	Sansys –Lbp 6020B	Mtma272571
P10	Ricoh	MP3350Aficio	FRHRO43547

For the Arabic document source identification problem, the content of the documents plays a significant role in determining the effectiveness of the identification process. Since printer forensics often relies on subtle variations in how specific characters are printed, the dataset must either focus on a fixed set of characters or be designed in a text-independent manner. In a text-dependent approach, the model is trained on specific characters or phrases that consistently appear across documents, such as common Arabic words or individual characters like "و" (waw). This approach may enhance the accuracy of identifying the printer source for those particular text elements but may be less generalizable to other document contents. In a text-independent approach, the character or word variations in the dataset are less important, focusing instead on the unique printer characteristics that emerge regardless of the document’s specific contents. This makes the model more robust, allowing it to generalize across various documents, regardless of the specific Arabic text.

The document is initially scanned at 1200 dpi with 8 bits per pixel in monochrome (grayscale) format, ensuring high-resolution and clear details. From the document, the Arabic character "و" (waw), set in 12-point Times New Roman font, is extracted as a separate image in JPG format. The dataset used for printer source identification includes text-independent images, meaning the character "و" is consistently extracted from various documents irrespective of the document’s content (free content). In the process of identifying the printer source, 200 images are randomly selected from the same dataset to serve as a test set representing 20% of the total images used for evaluation. The training dataset consists of 800 distinct images of the Arabic character "و" (waw), printed by various printers which makes up 80% of the data used in the model development. These training images capture the subtle variations in printing characteristics such as noise, texture, and alignment across different printer models. By using a diverse training set and a randomly selected test set, the model is evaluated on its ability to generalize printer-specific features, ensuring robust identification of the source printer based on minute inconsistencies or artifacts unique to each printer’s output.

This 80–20% split ensures that the model has access to a significant amount of data during training, allowing it to learn the subtle variations in printing characteristics such as noise, texture, and alignment across different printers. Meanwhile, the 10% test set provides a robust assessment of the model’s ability to generalize these learned features and accurately identify the source printer when presented with new, unseen images. The split ensures that while the model is well-trained, it is also thoroughly tested to avoid overfitting and to ensure it performs reliably in real-world scenarios. For the estimation of recognition results, accuracy was selected as an objective metric.

### Experimental protocol

For Arabic document source identification experiments, a clear and structured experimental protocol is essential to ensure reliable and replicable results. Here’s a step-by-step protocol:

### Dataset preparation

- Document Collection: Collect printed Arabic documents from a diverse set of printers. Aim to include a variety of printers (laser, inkjet, etc.), brands, and models to cover a wide range of potential sources. Ensure the documents contain a mix of text-independent content (e.g., randomly generated sentences, varying font styles and sizes).
- Scanning and Preprocessing: Scan each document at 1200 dpi in grayscale (8 bits per pixel) to capture fine details. Extract specific characters or words (e.g., "و" (waw)) from each document in a consistent format (e.g., 12-point Times New Roman font). Ensure the extracted images are saved in JPG format. Apply standard preprocessing techniques to each image, such as binarization, noise reduction, and alignment.
- Dataset Split: Create a balanced dataset with images of the extracted Arabic characters from different printers. Split the dataset into 80% training and 20% testing subsets. Ensure random selection of training and test images to maintain diversity across printers in both subsets.

### Model selection and experimental setup

- Baseline Model: Use traditional KNN with distance metrics such as Euclidean or Manhattan distance for baseline comparisons. Optimize the number of neighbors (K) through cross-validation using the training set.
- QKNN Setup: Implement Quantum k-Nearest Neighbors (QKNN), leveraging quantum algorithms to optimize the selection of K and accelerate the process. Prepare a quantum simulator for testing QKNN on the same dataset.
- Other Classifiers: Test additional machine learning models like SVM, Random Forest, or CNNs for comparative analysis.

### Evaluation metrics

- Accuracy: Measure the percentage of correctly identified printer sources.
- Confusion Matrix: Provide a confusion matrix to visualize printer source identification performance, particularly for closely related printers.

### Experimental phases

- Phase 1: Baseline Evaluation with Traditional KNN: Train a traditional KNN classifier using the training dataset. Conduct cross-validation on the training set to determine the optimal value of K. Evaluate the model on the test set, recording the accuracy
- Phase 2: Evaluation with QKNN: Train and evaluate the QKNN model using the same training and test sets. Compare the results (accuracy) with traditional KNN.
- Phase 3: Comparative evaluation with other models: Train and evaluate other classifiers like SVM or CNN using the same dataset split. Compare the results across all models.

### Analysis and discussion

- Accuracy and Efficiency Comparison: Analyze the performance difference between traditional KNN and QKNN, focusing on both accuracy and speedup in the process of selecting the optimal K.
- Evaluate how the model performs for closely related printers or more distinct printers.
- Impact of Dataset Size and Dimensionality: Examine how QKNN handles the dataset as the feature space or the number of samples grows, particularly focusing on the benefits of quantum algorithms in mitigating the curse of dimensionality.
- Generalization: Test the model’s generalization ability by introducing unseen printers to evaluate its robustness in real-world scenarios.

### Experimental results

The first set of experiments evaluates the classification accuracy of each printer by employing the optimal feature set, which ranges from approximately 5 to 7 features, as opposed to the preliminary 22 features. In our model, an optimal feature selection procedure is implemented using GA to identify the most significant features that contribute to a reduction in the overall assessment time while maintaining accuracy^40,41^. Table 4 illustrates the confusion matrix for identification with optimal feature set. The correct classification is revealed by the diagonal element, while the incorrect classification is revealed by the remaining elements. Additionally, Table 5 illustrates the comprehensive confusion matrix for identification with full descriptors.

**Table 4:** A confusion matrix using optimal descriptos.

	P1	P2	P3	P4	P5	P6	P7	P8	P9	P10
P1	10	0	0	0	0	0	0	0	0	0
P2	0	10	0	0	0	0	0	0	0	0
P3	0	0	9	1	0	0	0	0	0	0
P4	0	0	1	9	0	0	0	0	0	0
P5	0	0	0	0	9	1	0	0	0	0
P6	0	0	0	0	1	9	0	0	0	0
P7	0	0	0	0	0	0	10	0	0	0
P8	0	0	0	0	0	0	0	10	0	0
P9	0	0	0	0	0	0	0	0	10	0
P10	0	0	0	0	0	0	0	0	0	10
Accuracy	100	100	90	90	90	90	100	100	100	100

**Table 5:** A confusion matrix using all descriptos.

	P1	P2	P3	P4	P5	P6	P7	P8	P9	P10
P1	10	0	0	0	0	0	0	0	0	0
P2	0	10	0	0	0	0	0	0	0	0
P3	0	0	9	1	0	0	0	0	0	0
P4	0	0	1	9	0	0	0	0	0	0
P5	0	0	0	0	9	1	0	0	0	0
P6	0	0	0	0	1	9	0	0	0	0
P7	0	0	0	0	0	0	10	0	0	0
P8	0	0	0	0	0	0	0	10	0	0
P9	0	0	0	0	0	0	0	0	10	0
P10	0	0	0	0	0	0	0	0	0	10
Accuracy	100	100	90	90	90	90	100	100	100	100

It is evident from these tables that the proposed system exhibits superior accuracy in a significant number of printers, particularly those with model numbers P_3_ to P_8_, as some of these printers differ solely in their serial numbers and model numbers. This demonstrates the efficacy of the proposed system in extracting an optimal descriptor set that can precisely separate the texture features of the printer’s documents. Contrast, similarity, mean, diagonal moment, and the sum of variance features extracted from GLCM comprise the optimal set of features that collectively accomplish the highest level of accuracy, as determined by numerous experiments. In high dimensions, the distance between data points tends to concentrate, making it difficult to distinguish between different points. An optimal feature set reduces this problem, making distance measures more meaningful. Furthermore, by removing less important features, the model becomes less complex and less likely to overfit the training data^44^.

The second set of experiments was conducted to compare the identification accuracy of the suggested system, which employs GA to regulate the optimal descriptors and QKNN for classification, and the re-implemented printer identification system introduced in^6^ that utilized CNN, using the same data sets. The results indicate that the use of the five optimal features in conjunction with a QKNN with *k* = 3 classifier results in a 13% increase in the identification ratio compared to the same method without a feature selection phase (22 features with the same classifier), and a 3% improvement compared to the method that relies on CNN (see Table 6). The performance improvement is the result of the correct identification of printers, which is achieved by utilizing GA to extract optimal features (discriminative features) with the assistance of the multi-objective fitness function, which combines the recognition error and cardinality of the selected features.

**Table 6:** Comaprtive study.

Method	Accuracy rate (%)
Suggested method with optimal descriptors	96
Suggested method with all descriptors	83
Printer Identification method using CNN^45^	93
Printer Identification method using niching GA^46^	92

In general, the suggested model that follows feature-based classifiers typically require less computational power and memory compared to CNNs, which can be computationally intensive due to their deep layers and large number of parameters. Feature selection can lead to faster training times since the model only needs to learn from a reduced set of features rather than processing entire images through multiple layers of convolutions. CNNs generally require large amounts of labeled data to perform well. In scenarios where data is limited, feature selection methods can outperform CNNs because they don’t overfit as easily and can still extract meaningful patterns from a smaller dataset. Finally, GA can adaptively find features that are particularly suited to the small dataset, whereas CNNs may struggle to learn effective feature representations with limited data^6,12,19,35^.

Even with an optimal feature set, the choice of classifier plays a crucial role in enhancing recognition accuracy. The work presented in^46^ employs optimal printer’ descriptors with a traditional KNN classifier. Classical KNN suffers from the curse of dimensionality, where the distance metrics become less informative as the number of dimensions increases. QKNN, utilized by our model in conjunction with optimal printer’ descriptors, can mitigate this by efficiently handling high-dimensional data through quantum algorithms designed for high-dimensional spaces; and this explains the superiority of the proposed system over the compared one in terms of accuracy rate.

**Fig. 7:**
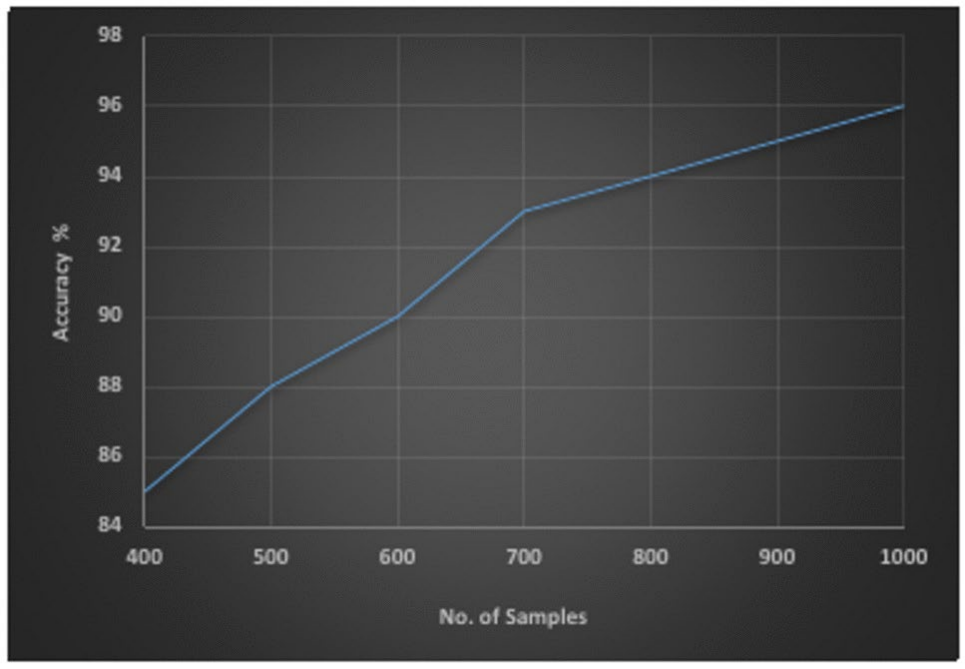
The correlation between the identification rate of the proposed recognition system and the total number of samples per printer.

The relationship between classification accuracy and the number of samples per class is an important aspect of machine learning and pattern recognition. Generally, classification accuracy tends to improve with an increase in the number of samples per class. With more samples per class, the training data can better capture the variability and nuances of each class. This comprehensive representation helps the classifier learn more accurate decision boundaries. Furthermore, more samples can help reduce overfitting, as the model is less likely to memorize the training data and more likely to generalize well to unseen data^45^. The third set of experiments aimed to illustrate the correlation between the identification rate of the proposed recognition system and the total number of samples per printer. It was observed that as the printer’s collection of registered instances increases, the likelihood of accurate identification also increases. As anticipated, the recognition rate in Fig. 7 rises as the number of samples grows because to the heightened inter-class diversity of printers. The accuracy ratio increases by around 3% for every additional 100 samples of cases in the printers’ dataset after reaching 400 cases.

The choice of the Arabic letter "و" (waw) for printer identification in major research studies can be attributed to several practical and technical reasons: (1) "و" is a common letter in Arabic, appearing frequently in various texts. This makes it a reliable candidate for analysis because it’s likely to be present in most documents. (2) The letter "و" has a distinctive and relatively simple shape. Unlike other letters, it is less prone to variations caused by handwriting or different font styles, making it easier to identify and analyze consistently. (3) "و" is a relatively simple character without complex strokes or connections to other letters in most cases. Its simplicity reduces the complexity of character recognition and segmentation. (4) In Arabic script, letters can have different forms depending on their position in a word (initial, medial, final, or isolated). "و" typically retains a consistent form regardless of its position, which simplifies the identification process. (5) Unlike many other Arabic letters, "و" rarely forms ligatures (connected forms) with other letters. This makes it easier to isolate and analyze printed text, reducing the potential for errors in segmentation. In order to verify that the character "Woo-و" is the most suitable letter for printer identification in the Arabic language, a fourth set of experiments was done, and the results are shown in Table 7. Typically, the "و" character comprises several bends and circles that may be used to derive a distinct collection of attributes capable of identifying each printer.

**Table 7:** Relationship between accuracy rate and letter type.

Letter	Accuracy (%)
Alef “أ”	79
Sad “ص”	74
Ain “ع”	81
Woo “و”	95

**Fig. 8:**
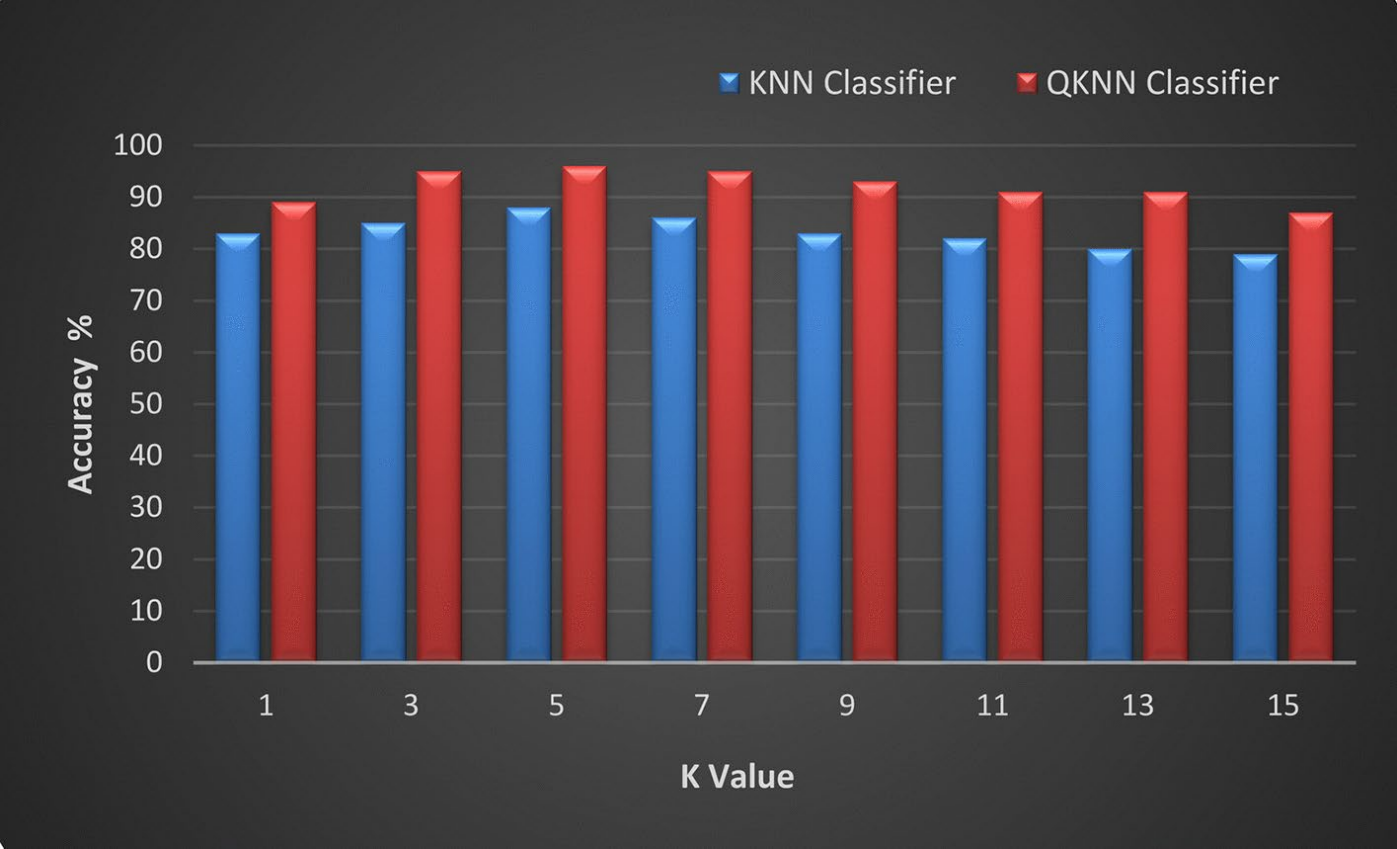
A comparative study between QKNN and KNN with different K’ values in terms of accuracy.

By conducting the last set of experiments, we can empirically verify the superiority of QKNN over traditional KNN for printer identification based on both accuracy and time complexity. QKNN’s potential for leveraging quantum computing resources should ideally demonstrate better accuracy and computational efficiency, especially as K increases, due to its ability to handle high-dimensional data more effectively, as revealed in Fig. 8. For higher values of *K*, QKNN can evaluate a larger set of nearest neighbors based on Euclidean distances in high-dimensional spaces. This capability is crucial for tasks like printer identification where a broader analysis of similarities and differences across multiple features is required. KNN-based classifier’s accuracy starts lower but increases as *K* increases from 1 to 5. This is because a small *K* makes KNN very sensitive to noise in the data, leading to overfitting; while QKNN shows higher accuracy compared to KNN for the same K values. Quantum algorithms can better handle the complexities in the feature space, leading to improved accuracy even at lower *K* values. Accuracy peaks around K = 5 and then slightly decreases. This is because, with higher *K*, KNN starts to include more distant neighbors, which may introduce irrelevant information. QKNN maintains its performance better than KNN as *K* increases. The quantum nature of QKNN allows it to handle the inclusion of more neighbors without a significant drop in accuracy. For KNN, accuracy continues to decrease slightly as *K* becomes larger. The classifier starts to underfit as it becomes too generalized, smoothing out the decision boundaries too much. QKNN still shows a slight decline in accuracy but remains higher than KNN. Quantum processing provides a more robust mechanism to deal with larger *K* values, maintaining better performance. For *K* = 3, the QKNN classifier outperforms the KNN classifier in terms of time. The QKNN classifier takes 230 ms, while the KNN classifier takes 300 ms. This suggests that the QKNN classifier is not only more accurate but also faster in execution, making it more efficient for real-time or time-sensitive applications, especially when using a moderate number of neighbors like *K* = 3.

The comparative results indicate that QKNN consistently outperforms KNN across different values of *K;* this is because QKNN leverages quantum superposition and entanglement to process multiple possibilities simultaneously, leading to more efficient and accurate neighbor identification. Classical KNN struggles with high-dimensional data due to the curse of dimensionality, where the distance metrics become less meaningful. Quantum computing can handle high-dimensional spaces more efficiently, mitigating this issue. Quantum parallelism enables the simultaneous calculation of Euclidean distances between the query point and multiple data points, drastically speeding up this process compared to classical computation. Finally, the potential for real-time processing of large datasets makes QKNN suitable for applications such as real-time data analysis and decision-making^15,47,48^.

### Evaluating the scalability and generalization of the suggested Arabic document source identification

The challenge of dataset availability for Arabic document source identification poses several key limitations affecting scalability and generalizability. Compared to other languages, Arabic suffers from a lack of diverse and comprehensive datasets, limiting the ability to model different fonts, printing methods, and printers. The complexity of Arabic script, with its cursive connections, diacritical marks, and varied fonts, adds to the challenge, as small datasets may fail to capture this variability. Limited printer variety and data scarcity lead to a higher risk of overfitting, where models perform well on training data but struggle to generalize. Additionally, collecting and annotating Arabic documents is labor-intensive, requiring collaboration with various institutions, and there is a lack of standardized datasets and benchmarks for Arabic document source identification.

Data augmentation can help address the challenges of using a small dataset for Arabic Document Source Identification by artificially increasing the diversity and volume of training data. Through techniques such as geometric transformations, noise addition, and style variation, augmentation simulates different printing conditions, fonts, and distortions, allowing models to learn from a broader range of examples. This reduces the risk of overfitting by enhancing the model’s ability to generalize to unseen data and improving robustness in identifying various printer sources. Additionally, augmentation can compensate for the lack of real-world data, bridging the gap between limited datasets and the complexity of real-world scenarios in forensic applications^49^. To implement data augmentation techniques, several software tools and libraries can be used effectively. Python-based libraries like TensorFlow, Keras, and PyTorch offer built-in augmentation functions such as rotation, scaling, and translation. For more custom and specific augmentations, especially for document noise injection and font style variations, OpenCV is a powerful library that allows for advanced image manipulation, such as adding noise, blurring, or adjusting contrast and brightness. Augmentor is another Python package tailored for image augmentation, providing a user-friendly API for geometric transformations and pixel-level alterations. These tools make it easier to implement and fine-tune augmentation processes to increase dataset diversity and improve model generalization.

The experiment aims to evaluate the scalability and generalization of an Arabic Document Source Identification model (printer identification) by comparing its performance on a small, traditional dataset (1,000 images) with an augmented dataset (10,000 images). The original dataset consists of Arabic-printed documents from 10 printers, and data augmentation techniques such as geometric transformations, noise injection, and font style variation are applied to expand the dataset. QKNN-based classification model will be trained on both datasets under the same conditions, with an 80/20 train-test split, consistent training epochs, batch size, and learning rate, to assess whether augmentation improves model accuracy and robustness in real-world scenarios. The results shown in Table 8 reveal that the model trained on the augmented dataset (Group 2) achieved slightly lower training accuracy (97%) but higher test accuracy (97.5%) compared to the model trained on the traditional dataset (Group 1), which had 98% training and 96% test accuracy. The improved test accuracy in Group 2 can be attributed to the data augmentation techniques, which introduced more diversity (through geometric transformations, noise, and font variations), allowing the model to learn more generalizable features. This helped the model perform better on unseen data. Both groups prevent overfitting, as the gap between training and test accuracy is small. In Group 1, the simplicity of the smaller dataset limits overfitting, while in Group 2, the augmented dataset’s variability helps the model avoid memorizing the training data and instead focus on generalization.

**Table 8:** Accuracy comparison of traditional vs. augmented datasets for Arabic document source identification with optimal descriptors.

Experimental group	Dataset size	Training accuracy (%)	Test accuracy (%)	Overfitting
Group 1 (Traditional dataset)	1,000 images	98	96	No
Group 2 (Augmented dataset)	10,000 images	97	97.5	No

Furthermore, the results in Table 9 demonstrate that incorporating data augmentation consistently improves the accuracy across all methods including both the proposed approach and traditional machine learning models. In this case, a variety of classifiers—including Convolutional Neural Networks (CNN), Support Vector Machines (SVM), Random Forests (RF), Decision Trees (DT), and K-Nearest Neighbors (KNN)—are employed as comparative models. Each classifier is used as a "black box," meaning that they operate with their default configurations without extensive parameter tuning. These models are then tested in place of the QKNN module within the system. The goal is to evaluate how each of these traditional classifiers performs relative to the QKNN in terms of accuracy, in the task of printer identification using the Arabic character "و" with and without data augmentation technique. For instance, the suggested method with optimal descriptors achieves an accuracy increase from 96% to 97.5%, while traditional machine learning models like SVM, Random Forest, and k-NN show even more significant improvements (e.g., SVM jumps from 85 to 93%). This enhancement is due to data augmentation’s ability to artificially expand the dataset by introducing new, diverse variations of the original data. This diversity enables models to generalize better, capturing more intricate patterns and reducing overfitting, which is especially critical when the original dataset is small. Consequently, the augmented dataset helps all models improve their learning capabilities and ultimately achieve higher accuracy rates.

**Table 9:** Accuracy rate (%) comparison of printer identification methods using original and augmented datasets.

Method	Original dataset	Augmented dataset
Suggested method with optimal descriptors	96	97.5
Suggested method with all descriptors	83	87
Printer identification method using CNN	93	96
Printer identification method using niching GA	92	95
Support vector machine (SVM)	85	93
Random forest (RF)	82	92
Decision trees (DT)	77	90
k-Nearest neighbors (k-NN)	83	94

## Conclusions

In forensic analysis, document verification identifying the specific printer that produced a document is crucial. Traditional methods of printer identification rely on classical algorithms and manual inspection, which can be time-consuming and less accurate when dealing with large datasets or high-dimensional feature spaces. This process involves analyzing unique characteristics left by printers, such as print artifacts, texture features, edge and contour features, and noise patterns. However, classical KNN algorithms may struggle with efficiency and scalability, particularly when processing high-dimensional data or large datasets. Combining an optimal feature set with QKNN offers a promising solution to address these challenges. Computing Euclidean distances across large datasets and for higher values of *K* can be computationally intensive. QKNN’s quantum speedup allows for faster and more efficient distance computations, leading to quicker identification and classification of printer outputs based on their unique characteristics.

In the suggested model, utilizing GLCM for feature extraction in printer identification provides a robust and detailed analysis of the textural properties of printed documents. This helps in accurately distinguishing between different printers, making it a valuable tool in forensic document examination and printer identification applications.

The suggested method with optimal descriptors achieved the highest accuracy (96%), demonstrating the effectiveness of selecting key features for printer identification. When using all descriptors, the accuracy dropped to 83%, indicating that including irrelevant or redundant features can reduce model performance. The CNN-based approach performed well, reaching 93%, while the niching Genetic Algorithm (GA) method closely followed with 92%, showcasing their competitive abilities. Traditional classifiers such as SVM, RF, DT, and k-NN underperformed compared to more advanced methods, with accuracies ranging from 77 to 85%, highlighting the advantages of feature optimization and deep learning techniques. Future work includes conducting large-scale tests to evaluate the performance of QKNN with extensive datasets and in various practical scenarios. Investigating other quantum algorithms and their potential applications in printer identification and other forensic tasks. Enhancing feature extraction techniques using deep learning and other advanced methods to improve the robustness and accuracy of printer identification.

## Data Availability

The data that support the findings of this study are available from the corresponding author, Saad M. Darwish, upon reasonable request.
